# Magnetic states of nanostructures containing Ni^2+^ ions at the surface of SiO_2_ nanospheres

**DOI:** 10.1038/s41598-017-11394-8

**Published:** 2017-09-07

**Authors:** Gabriele Barrera, Gabriele Alberto, Paola Tiberto, Gianmario Martra, Paolo Allia

**Affiliations:** 1 I.N.Ri.M., Nanoscience and materials, Strada delle Cacce 91, 10135 Torino, Italy; 2 Department of Chemistry and NIS Centre, University of Torino, via Giuria 7, 10125 Torino, Italy; 3 Department of Applied Science and Technology, Polytechnic of Torino, Corso Duca degli Abruzzi 24, 10129 Torino, Italy

## Abstract

Ultra-small magnetic particles containing Ni^2+^ ions were grown at the surface of SiO_2_ spheroidal nanoparticles (typical diameter: 50 nm) starting from NiCl_2_ solutions. Depending on preparation details, two samples characterized by magnetic sub-nanostructures or lamellar sub-nanoparticles at the SiO_2_ nanosphere surface were obtained. The decorated SiO_2_ nanospheres were submitted to physico-chemical and magnetic characterization. In both samples, a magnetically blocked phase is observed at low temperature. Below 5 K, discontinuities in isothermal magnetization loops and magnetic relaxation effects suggest the onset of coherent quantum tunneling of nanoparticle magnetization (QTM). Relaxation effects give are described by a field- and temperature-dependent magnetic viscosity S_V_(H,T); the total spin number of magnetic units is estimated by fitting the isothermal S_V_(H) curve to a model for an assembly of particles with random anisotropy axes. The mean number of aligned spins involved in the low-temperature relaxation is 32 and 15 in the two considered samples. Phonon-assisted QTM plays an increasingly important role with raising temperature and the quantum regime gradually merges with the classical behavior. Above the blocking temperature the magnetic units behave as classical superparamagnetic particles. When the intra-particle ferromagnetic order disappears the Ni^2+^ ions respond individually to the magnetic field.

## Introduction

Magnetism has been greatly influenced in his foundations and applications by the advent of magnetic nanoparticles, currently prepared by many efficient techniques^1–4^.

Magnetic nanoparticles are often inserted in a diamagnetic host, either fluid or solid; when the particles themselves are first obtained as a powder and subsequently embedded in a solid^5^, aggregation is a highly probable event; some fraction of magnetic aggregates is typically found in these nanocomposites, even at small filler concentrations^6^. Nanoparticle aggregation has in turn pros and cons from the viewpoint of applications^7^; on the other hand, the physical interpretation is complicated by the coexistence of an individual magnetic response of isolated nanoparticles and a collective response from aggregates.

Alternatively, nanoparticulate systems are prepared by bottom-up techniques allowing the magnetic nanoparticles to nucleate and grow either simultaneously to the development of specific mesostructures in the host matrix (a typical example being magnetic carbon nanotubes^8^) or in the presence of preformed diamagnetic nanostructures. In both cases, the meso/nanostructures constituting the host material can be decorated at their surfaces by magnetic nanoparticles; the decorated structures display novel functional properties whilst the tendency to aggregation among nanoparticles is naturally reduced, allowing a more accurate description of magnetic phenomena to be given.

In this work, two nanosystems composed of SiO_2_ nanospheres decorated by ultra-small magnetic particles rich in Ni^2+^ ions (having at least one dimension of the order of 1–2 nm) have been prepared by two slightly differentiated chemical routes, inspired by methods set up for the preparation of Ni supported on silica heterogeneous catalysts^9, 10^. In recent years, silica has been widely used both as core^11^ and shell^12^ in magnetic nanoparticles, or in double-layered, sandwiched systems^13^. Our ultra-small, magnetically active units will be thereafter termed sub-nanoparticles or sub-nanostructures, depending on the material considered, as explained later. Both nanomaterials have been submitted to physicochemical characterization at room temperature and to detailed magnetic characterization from 2 to 300 K.

Exciting properties emerge at low temperature when the size of a three-dimensional array of spins is below some threshold. One fascinating aspect in studying ultra-small magnetic particles is the boundary between quantum and classical effects. In single molecule magnets a full quantum-mechanical approach is the key to interpret a variety of distinctive phenomena related to coherent tunneling of magnetization, such as reversible steps in isothermal hysteresis loops and the low-temperature relaxation of the magnetization (also called magnetic viscosity). Effects related to resonant or phonon-assisted tunneling of magnetization are not solely a feature of single molecule magnets, having been observed as well in magnetic clusters^14^, ultra-small particles^15^ and novel functional materials containing magnetic ions^16^. Atomic-scale magnetic systems such as high anisotropy metallic adatoms on a substrate^17, 18^ provide the ultimate example of ultra-small magnetic units described by a full quantum approach.

On the other hand, when the size of the organized magnetic units is much larger than a few nanometers a standard classical picture applies. However, the boundary between quantum and classical effects depends not only on particle size but also on temperature, so that a single well-prepared and well-characterized magnetic system can provide a unique opportunity to get a comprehensive picture of the transition from a quantum to a classical magnetic regime when the temperature is increased.

The heterogeneous nanosystems studied in this work will prove to be suitable playgrounds not only to study low-temperature phenomena arising from quantum tunneling of magnetization, but also to follow the continuous evolution of the magnetic order as the temperature is gradually increased.

The strict analogies existing between the low-temperature behavior of these ultra-small magnetic particles and single molecule magnets allow us to infer that the two systems have common potential applications in quantum computing and information technologies, where the requirement of extreme miniaturization is a major issue^19^. Although the magnetic response of ultra-small particles is less sharp than the one of single molecule magnets, the chemical route exploited in the production of SiO_2_ nanospheres decorated by magnetic sub-nanostructures/sub-nanoparticles is easier and cheaper and may lead to larger amounts of end products.

## Results and Discussion

### Physico-chemical characterization of Ni^2+^-SiO_2_ NPs

**Figure 1:**
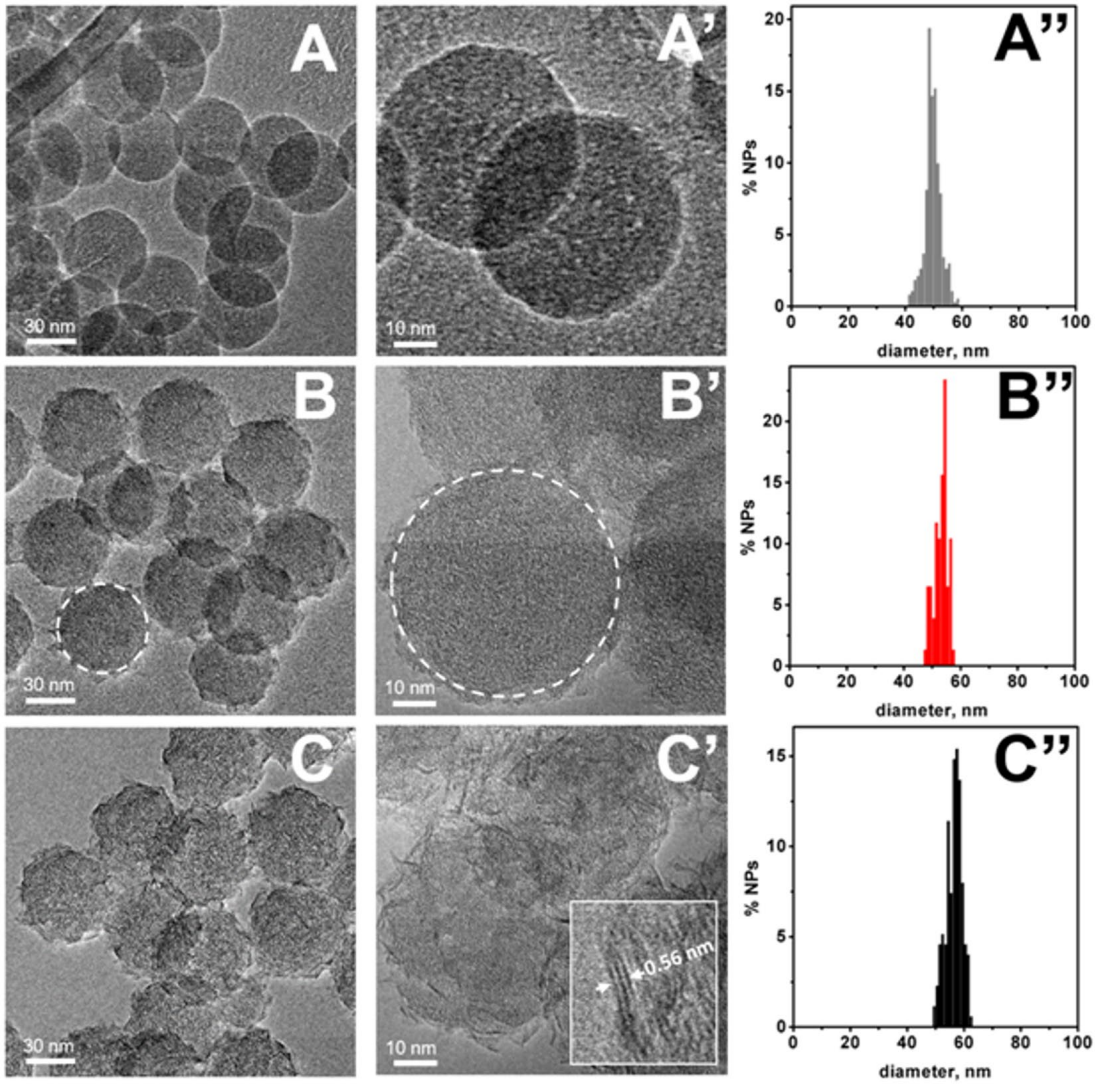
Representative HRTEM images and size distribution histograms of spheroidal NPs of: (**A–A”**) bare SiO_2_, (**B–B”**) sample 1, and (**C–C”**) sample 2 nanoparticles.

A synoptic view of HRTEM images of bare SiO_2_ NPs (reported for the sake of comparison), sample 1 and sample 2 Ni^2+^- SiO_2_ NPs and related particle size distributions is shown in Fig. 1.

It can be observed that the adopted preparation method resulted in the formation of spherical bare silica NPs, quite regular in shape and exhibiting a narrow size distribution with a diameter mean (d_m_) value of 50 ± 3 nm (panels A–A”). The spherical morphology was retained also by SiO_2_-based NPs added with Ni^2+^, although with some increase in size of ca. 10% for sample 1 (d_m_ = 55 ± 4 nm) and ca. 16% for sample 2 (d_m_ = 58 ± 5 nm) (panels B–B” and C–C”, respectively). Noteworthy, the border of the 2D projection of sample 1 NPs (panels B, B’) are decorated by small sub-nanostructures, quite irregular in shape, with main and minor sizes, in the image plan, of few nanometers. In the case of sample 2, nanoparticles are coated by a quite dense texture of sub-nanoparticles (C, C’) appearing elongated in the projection on the image plan, with length and width in the 10–5 nm and 2–1 nm range, respectively. Moreover, in a number of cases these sub-nanoparticles exhibited a larger contrast with respect sub-nanostructures in sample 1, suggesting they should be thicker. In addition, lattice fringes 0.23 nm apart can be observed in images of sub-nanoparticles in sample 2 taken at further higher magnification (panel C’, inset).

HRTEM observations were augmented by EDX analysis; relevant data obtained for Ni-containing material are the following: the percentage of Ni atoms with respect to Si atoms Si/Ni (% at) is 5.3 (±0.2) · 10^−2^ and 6.0 (±0.2) · 10^−2^ in sample 1 and 2, respectively; the number of Ni atoms per gram of the sample (supposing a stoichiometry Ni_0.053_SiO_2_ and Ni_0.060_SiO_2_ for sample 1 and 2, respectively) is 5.3 (±0.2) · 10^20^ in sample 1 and 5.9 (±0.2) · 10^20^ in sample 2.

**Figure 2:**
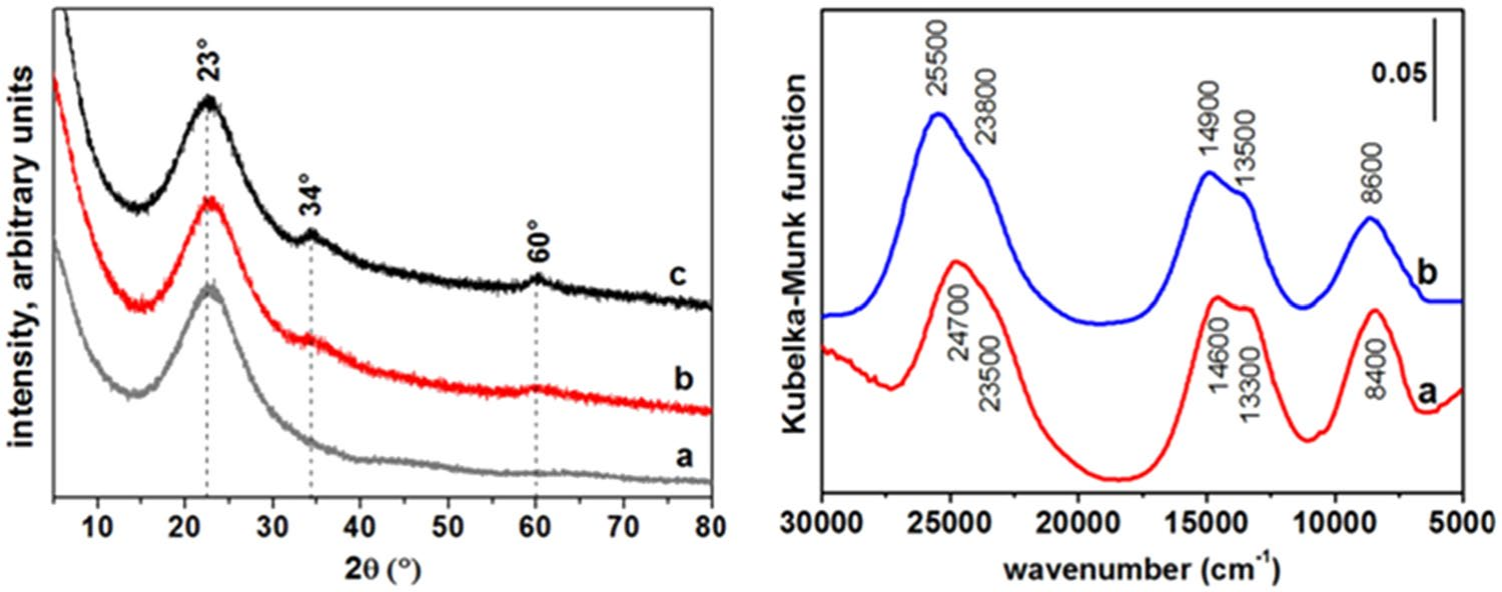
(**a**) XRPD patterns of: (**a**) bare SiO_2_ NPs, (**b**) sample 1 and (**c**) sample 2; (**b**). Diffuse reflectance UV-Vis-NIR spectra, in the 30000–5000 cm^−1^ range of (**a**) sample 1 and (**b**) sample 2, in air.

Structural insights on additional sub-nanoparticles present in Ni^2+^-SiO_2_ NPs samples 1 and 2 were obtained by X-ray powder diffraction (Fig. 2a). As expected, the pattern of bare SiO_2_ NPs (curve a) exhibits a broad reflection with maximum at ca. 23°, typical of amorphous silicas. Conversely, additional broad signals at ca. 34° and 60° are present in the pattern of Ni^2+^-SiO_2_ NPs, extremely weak for sample 1 (curve b) and still weak but better defined for sample 2 (curve c). They may be attributed either to the 10 and 11 reflections of a turbostratic nickel hydroxide, or to the 13–20 and 06–33 reflections of ill-crystallized 1:1 or 2:1 nickel phyllosilicates^9^. The signal at ca 34° is due to planes calculated to be ca. 0.26 nm apart, in a satisfactory agreement with the distance between lattice fringes observed in HRTEM images of sub-nanoparticles in sample 2 (by considering the relevant relative error when the distance it measured over few fringes). Moreover, the 001 line of nickel hydroxide or nickel phyllosilicate expected around 10° ^9^ is not visible, again in agreement with the thickness of sub-nanostructures along the[001] direction limited to few unit cells. Moreover, the broadness of d_hk_ signals in the patterns indicates they are due to coherent scattering domains rather limited in size.

Thus, the collection of HRTEM and XRPD data indicate that Ni atoms are dispersed in smaller, and likely almost amorphous sub-nanostructures in sample 1, and in larger layered ones, still very thin along the[001] direction, in sample 2. In both cases, the original Ni^2+^ oxidation state was retained, as proved by DR UV-Vis-NIR spectra (Fig. 2b), exhibiting a pattern due to d-d transitions typical of Ni^2+^ ions dispersed at the surface of silica (curve a)^20^ or in Ni(OH)_2_ (curve b)^21^ for sample 1 and sample 2, respectively. Thus, also optical spectra are in agreement with the higher dispersion of Ni cations at the surface of SiO_2_ nanoparticles in sample 1.

### Magnetic behavior

The magnetic loops measured in samples 1 and 2 between 2 and 300 K are shown in the Supplementary Information as Figure S1.

**Figure 3:**
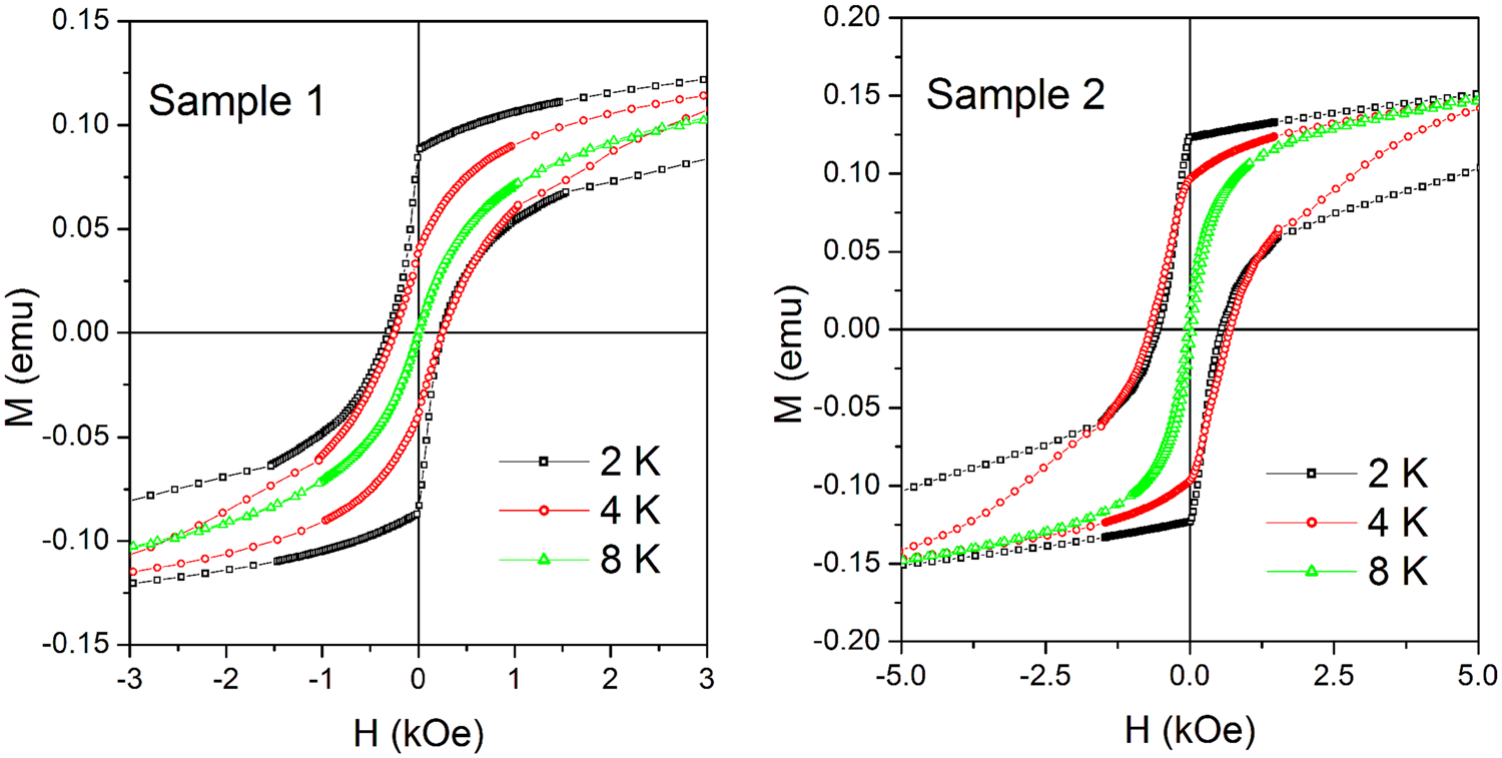
Low-field region of selected magnetic hysteresis loops in the range 2–8 K.

The low-field region of the M(H) curves below 10 K is shown in Fig. 3. An abrupt change of slope of M(H) with a discontinuous derivative is clearly observed at T = 2 K and H ≅ 0 on both loop branches. This is a first evidence of a change of regime taking place in both materials in the low-temperature limit.

**Figure 4:**
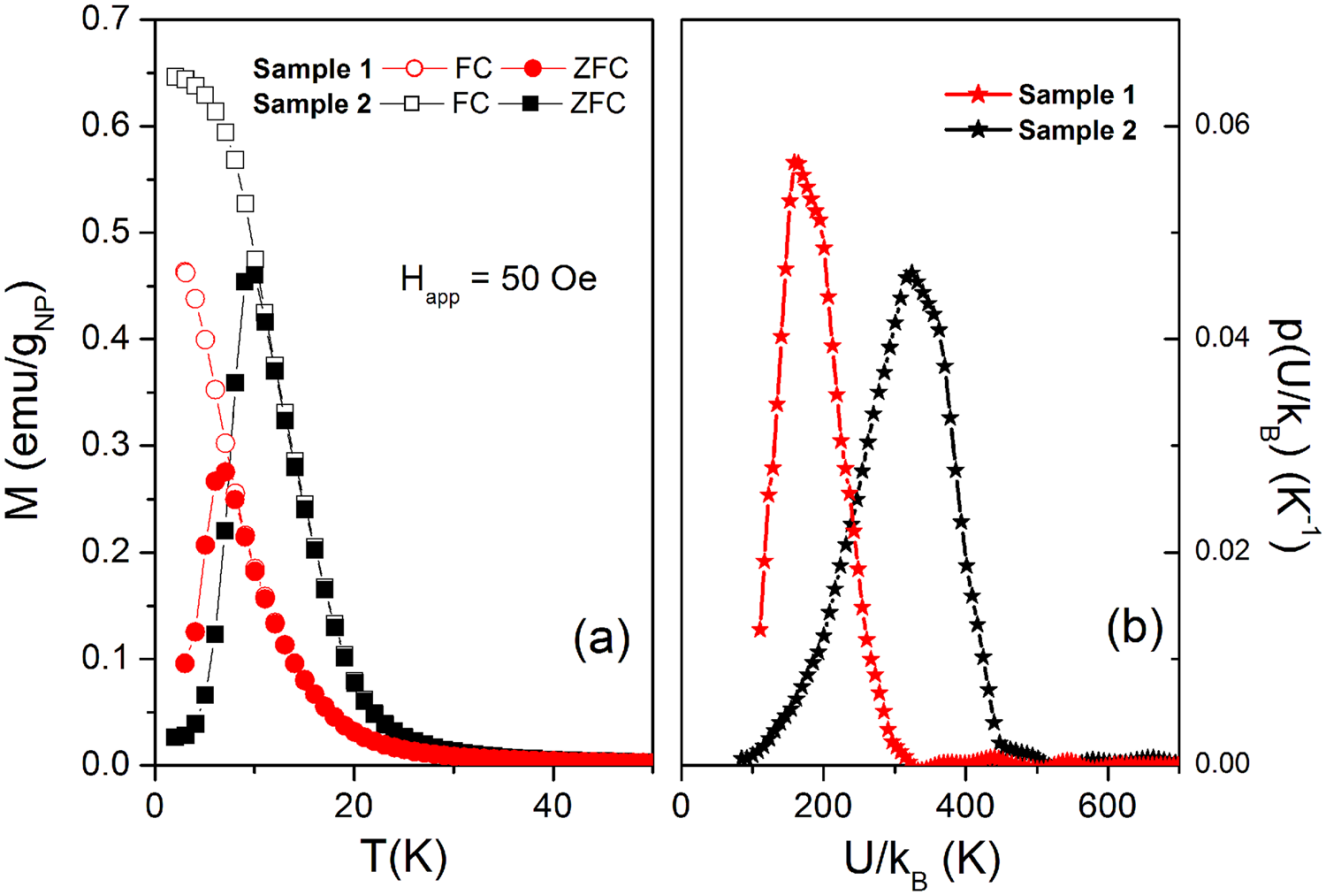
(**a**) FC/ZFC curves measured in samples 1 and 2; (**b**) estimated energy barrier distribution in samples 1 and 2 obtained from FC/ZFC curve analysis (see text for details).

The FC/ZFC curves of both samples measured under a field of 50 Oe are reported in Fig. 4(a). In both materials, the merging temperature of the curves is coincident with the peak of the ZFC curve, which occurs at T_B_ = 6.7 K in sample 1 and T_B_ = 9.7 K in sample 2.

The behavior is clearly related to the presence of ultra-small particles narrowly distributed in size which undergo single-particle blocking driven by an Arrhenius law of the type ${\rm{\upsilon }}={\upsilon }_{0}{e}^{-\frac{U}{{k}_{B}T}}$. In order to ascertain the values of attempt frequency ${\upsilon }_{0}$ and energy barrier U, AC measurements of the initial susceptibility have been conducted, as shown in Figure S2 of the Supplementary Information. The results are: ${\upsilon }_{0}$ = 1.45 × 10^13^ Hz, *U*_1_/k_B_ = 180 K for sample 1, ${\upsilon }_{0}$ = 2.68 × 10^16^ Hz, *U*_2_/k_B_ = 345 K for sample 2. As expected from morphology considerations, the energy barrier (which is thought to arise from magnetic anisotropy) is higher in the more anisotropic, laregr particles of sample 2. In both cases, ${\upsilon }_{0}$ turns out to be higher than expected on the basis of simple considerations^22^. Such “unphysical” values can be explained by invoking the Meyer-Neldel (MN) rule (or compensation law) which naturally arises when the activation energy for a kinetic process is significantly larger than both the typical excitations available (equilibrium-state phonons) and thermal energy k_B_T^23^, which is exactly the case here. According to the MN rule, when the activation energy increases the attempt frequency ${\upsilon }_{0}$ increases too^24^. In our case such a condition is satisfied, even if it can be somewhat misleading to compare values of ${\upsilon }_{0}$ and *U* obtained in different materials.

An estimate of the distribution of energy barriers p(*U*) around *U*_1_ and *U*_2_ has been obtained from ZFC/FC curve analysis according to a well-established technique^25^ and is shown in Fig. 4(b). In both samples, the distribution is quite narrow (the variance being slightly larger in sample 2); no energy barriers with *U*/k_B_ values below 100 K are found.

The appearance of sharp discontinuities of the M(H) curves measured around H = 0 at T = 2 K (see Fig. 3) bears close similarities with the observations in a variety of magnetic systems where the magnetization undergoes coherent quantum tunneling (QTM)^16, 26–28^. These include single molecule magnets^29–32^ as well as magnetic clusters^14^, magnetic nanoparticles^15, 33–35^, and magnetic domain walls^36, 37^. One of the distinctive features related to QTM is the relaxation of magnetization after a huge magnetic-field jump at temperatures where activation processes are wiped out. The relaxing magnetization usually follows a law of the type:

$${\rm{M}}({\rm{t}})={M(t}_{0})[1-{S}_{{\rm{V}}}({\rm{H}},{\rm{T}})\,\mathrm{ln}(\frac{{\rm{t}}}{{{\rm{t}}}_{0}})]$$

where t_0_ is a properly chosen initial time and the term S_V_(H, T) may be called the “magnetic viscosity”^26, 37, 38^.

In order to test our materials, we have performed magnetization relaxation experiments using a measuring setup characterized by the operating ranges and dynamic characteristics declared in the Materials and Methods section. The dynamic characteristics give rise to a finite stabilization time t_0_; relaxation processes h\aving time constants less than t_0_ are lost.

1) S vs. H measurements at fixed T

- the sample is brought to a temperature T_H_ well above the blocking/merging (irreversibility) temperature of FC/ZFC curves and a strong magnetic field (H_A_ = 70 kOe) is applied for 60 s; in our case, T_H_ is set to 20 K;
- the sample is brought down to a much lower temperature (T = 2 K = T_H_/10) under field H_A_. After thermal stabilization, H_A_ is suddenly changed to a lower value H (H takes values from +50 KOe, to 0);
- the magnetization is measured at T = 2 K as a function of time for 3600 s under the field H; after each measurement the sample is again brought to 20 K and the field H_A_ is applied as in (a);
- the entire procedure is repeated starting from H_A_ = −70 kOe (with H taking the values as in (b), with opposite sign).
  2) S_V_ vs. T measurements at fixed H
- following to pre-treatment as in (a), the sample is brought to the low temperature T still under the field HA. After thermal stabilization, HA is suddenly changed to H = 0;
- the magnetization is measured at H = 0 as a function of time for 3600 s at the temperature T; after each measurement the sample is again brought to 20 K and the field HA is applied as in (e); in a cycle of measurements the final temperature T takes values between 2 and 5.5 K (sample 1) or 8 K (sample 2), i.e. below TB in both cases.

**Figure 5:**
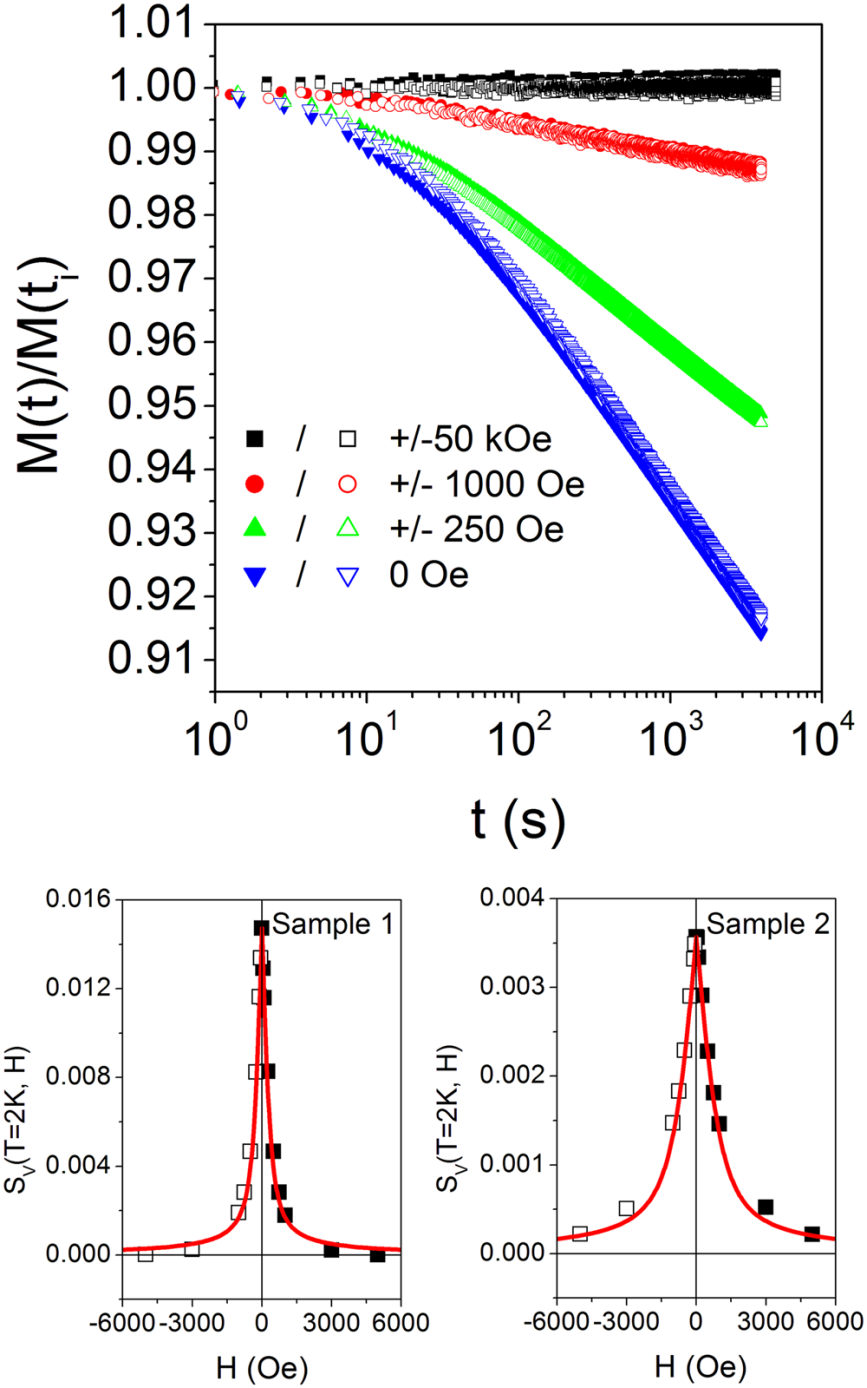
Top panel: relaxation of sample 1 at T = 2 K for different values of the applied field H (positive and negative) after pre-treatment at T_A_ = 20 K under the field H_A_ = 70 kOe (see text for details); bottom panel: magnetic field behavior of the magnetic viscosity S_V_(T = 2 K, H) for positive (full symbols) and negative H_A_ (open symbols) in samples 1 and 2. Red lines: best fit to Eq. (1) of Supplementary Information.

Typical relaxation curves taken in sample 1 at T = 2 K for different value of H are shown in Fig. 5 (top panel) (a closely similar behavior being measured in sample 2). The initial time t_i_ corresponds to the start of the measurement after stabilization of the field H_M_. Generally speaking, the magnetization under positive and negative fields (steps (c) and (d) above) develops a quasi-logarithmic relaxing behavior allowing one to accurately determine S_V_(T = 2 K,H); the effect is symmetric with respect to the inversion of H.

Such a relaxation, which occurs at a temperature well below the effective temperature for the activated barrier crossing (T_1_ ≡ *U*_1_/k_B_ ≅ 180 K; T_2_ ≡ *U*_2_/k_B_ ≅ 345 K) is consistent with the onset of coherent quantum tunneling of the magnetization.

The behavior of S_V_(H) at T = 2 K is reported in Fig. 5 (bottom panel) for both samples as a function of H for positive and negative H_A_. The effect is perfectly symmetric with respect to H = 0; the magnetic viscosity rapidly disappears with increasing |H|, the relaxation being inhibited by the magnetic field.

**Figure 6:**
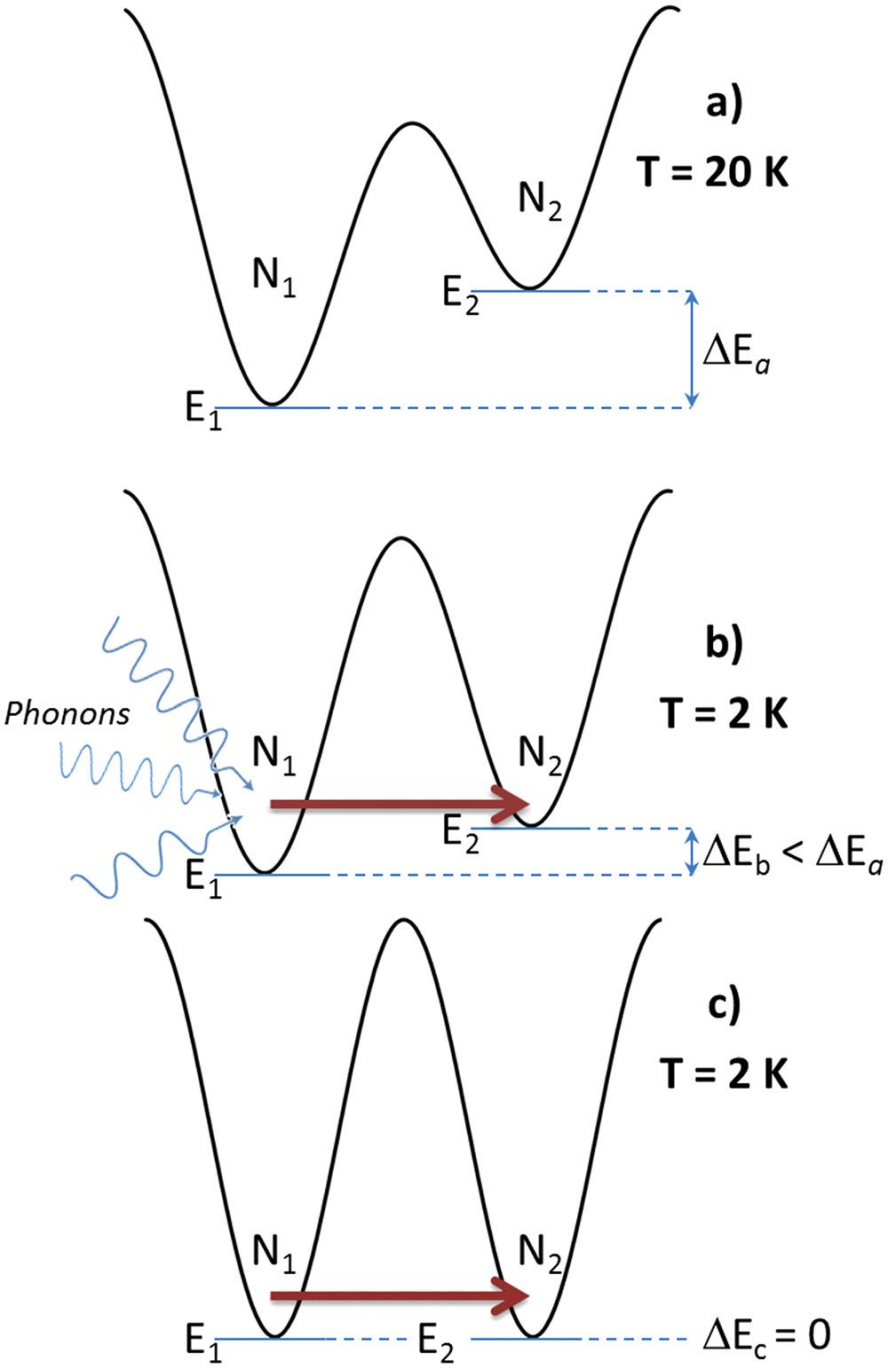
Behavior of a double-well system at different temperatures and different applied fields (see text). The sketch applies to particles with easy anisotropy axis parallel to the direction of the magnetic field.

This behavior can be understood in terms of a double-well scheme, as sketched in Fig. 6 (which applies for simplicity to particles whose easy axis is parallel the applied field): when the sample is kept at high temperature (T_A_ = 20 K, well above blocking) under the field H_A_ = +70 kOe, the energy difference ΔE_a_ = E_2_-E_1_ is large and a substantial imbalance of population in the two wells is created, so that N_1_» N_2_, (Fig. 6(a)). When the sample temperature is lowered to T and the field is removed, the double well becomes symmetric (ΔE_c_ = 0, Fig. 6(c)) and the equilibrium value of the N_1_/N_2_ becomes 1; however, the thermal relaxation rate predicted by the Arrhenius law at this temperature is so low that the N_1_/N_2_ ratio is blocked to the off-equilibrium value and no magnetic relaxation originating by thermal activation over the barrier should be measured in the explored time interval. It is therefore suggested that the observed relaxation originates from resonant quantum tunneling between the two minima which occurs at no energy cost, providing a mechanism for the redistribution of N_1_ and N_2_ between the two wells. Instead, when the field is decreased to a positive, nonzero value of H starting from large positive H_A_, the asymmetry of the double well is just reduced (ΔE_b_ < ΔE_a_, Fig. 6(b)); the decrease of the N_1_/N_2_ ratio towards the corresponding equilibrium value can only take place if the energy difference ΔE_b_ is transferred from the thermal bath to the tunneling system (basically, via an exchange of phonons).

The picture can be generalized to the case of a set of particles whose easy axes are randomly pointing towards all directions in space (see Supplementary Information). The red lines in Fig. 5 (bottom panel) are drawn matching Eq. (1) of the Supplementary Information to the value of S_V_(T,0) we actually measure in the two samples; the only free parameter is the magnetic moment μ per nanoparticle entering the expressions of α and β (see Supplementary Information), which can be therefore univocally determined: in sample 1, the mean magnetic moment taking part in coherent tunneling is estimated to be μ = 100 μ_B_, while μ = 36 μ_B_ in sample 2.

**Figure 7:**
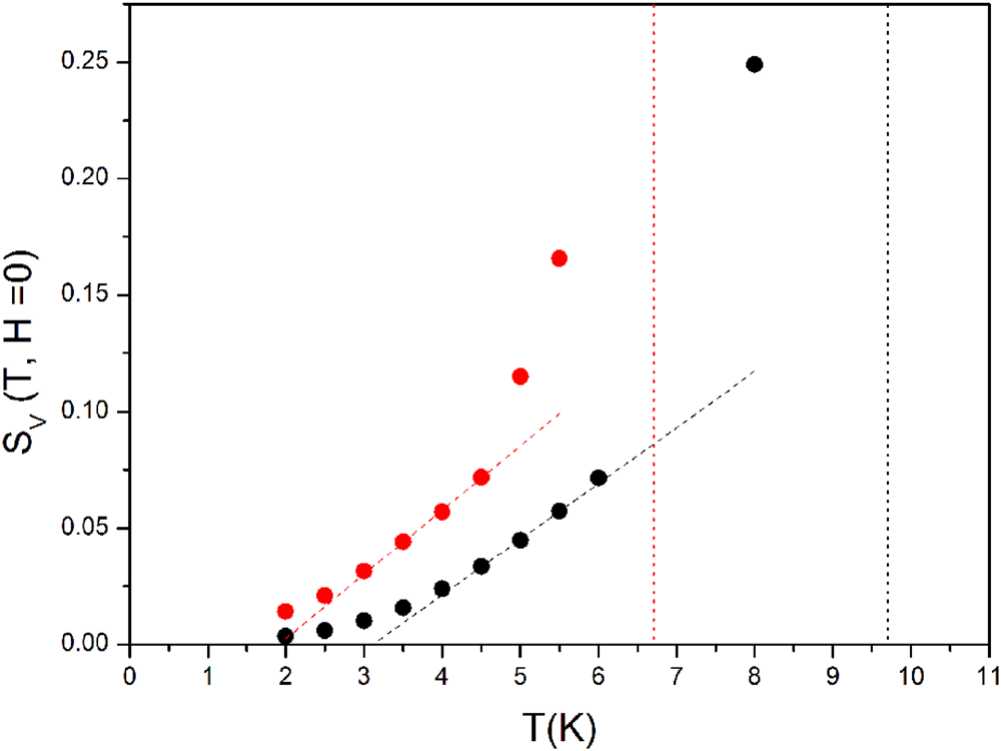
Temperature behavior of the magnetic viscosity S_V_(T, H = 0) for sample 1 (red symbols) and 2 (black symbols). Red and black dotted vertical lines mark the blocking temperatures. Dashed lines put in evidence the linear part of S_V_(T).

The behavior of S_V_(T) at H = 0 is reported in Fig. 7 for both samples. The curves are linear with T over a narrow temperature interval (dashed lines); substantial deviations from the straight line are observed both at low temperature and when T approaches T_B_ (marked by the vertical dotted lines)^39^. A linear dependence of magnetic viscosity on T is typically observed in nanoparticle systems below the blocking temperature^15, 40^. Such a linear behavior has been ascribed to thermally activated barrier crossing^41^; in this case, the relation ${{\rm{S}}}_{{\rm{V}}}({\rm{T}})\cong \frac{{{\rm{k}}}_{{\rm{B}}}{\rm{T}}}{\langle {\rm{U}}\rangle }$ applies, <U> being the average anisotropy barrier^41, 42^. In various blocked-nanoparticle systems the linear law does no longer hold below some threshold temperature and is suddenly replaced by a constant magnetic viscosity S_V_; this effect has been considered as the hallmark of the onset of quantum regime involving resonant tunneling between the double-well states of lowest energy^15, 40, 42^.

Actually, no temperature-independent relaxation of magnetization can be identified in our samples, the S_V_(T) curve just displaying a positive curvature instead. This can be understood in terms of QTM models^43, 44^ indicating that at sufficiently high temperatures quantum tunneling of large spins across the barrier U does not involve the fundamental level only, but includes significant contributions from excited states in the double well also^44, 45^, the larger tunneling probability counterbalancing the lower probability of the system being excited above the ground state. In fact, the resonant-tunneling amplitude increases by reducing the distance in energy of the resonant states from the top of the barrier^46^ so that thermal population of the higher excited states in the double well plays a central role in relaxation. The thermally-assisted tunneling regime must be taken into account when the properties of relaxing large-spin systems such as molecular magnets or magnetic clusters are described^31, 45^.

The non-linearity of S_V_(T) observed in our samples below 4 K can therefore be explained in terms of the concurrence of temperature-independent quantum tunneling and of a thermally activated contribution. In principle, the latter can arise from either thermal excitation over the barrier or phonon-assisted tunneling across the barrier; the experimental evidence indicates that the second picture is more appropriate to describe the magnetic relaxation in these systems.

In fact, the model of barrier crossing dominated by thermal transitions^41^ predicts the values $\frac{{k}_{B}}{{U}_{1}}\,=$ 4.0 × 10^−3^ K^−1^ and $\frac{{k}_{B}}{{U}_{2}}\,=$ 2.1 × 10^−3^ K^−1^ for the slope of the linear region of the S_V_(T) curve; the experiment however indicates that the magnetic relaxation is much stronger than predicted by the classical picture, with similar slopes in the linear region of the curve (see Fig. 7; the measured slopes are 2.7 × 10^−2^ K^−1^ and 2.4 × 10^−2^ K^−1^ for samples 1 and 2 respectively). Considering that in both samples the average barriers U_1_/k_B_ and U_2_/k_B_ are rather high (hundreds of K), thermal activation over the barrier would play a significant role in the temperature interval 2–4 K only by invoking the presence of a wide distribution of barriers with a substantial tail towards zero energy^26^; however, we observe a narrow distribution of barrier energies around U_1_/k_B_ or U_2_/k_B_ with no barriers below 100 K (Fig. 4(b)) and we can confidently rule out this possibility. It is concluded that the leading contribution to magnetic viscosity in these samples derives from phonon-assisted, quantum-mechanical tunneling through the barrier; in our opinion, thermal activation over the barrier plays a minor role below 4 K.

Finally, our measurements show that S_V_(T) is enhanced more than linearly as T approaches T_B_; this is in agreement with the peak of S_V_ at T_B_ observed by Ibrahim *et al*.^39^ who suggested a sort of critical behavior of S_V_ around the blocking temperature.

Relaxation of M sustained by quantum tunneling is responsible for the singular features observed in the M(H) loops of the samples at 2 K (Fig. 3). When H is slowly decreased from H_Max_ in the upper branch of the loop, relaxation effects do not play any significant role until H is reduced to about zero; there, a substantial relaxation of M begins to occur and is responsible for the sudden change in slope we observe in both materials; however, in this measurement H keeps changing at 2,5 Oe/s around H = 0 (its absolute value starts increasing again on the negative side) so that the actual shape of the curve reflects the convolution of the dynamic loop with the relaxation effect.

**Figure 8:**
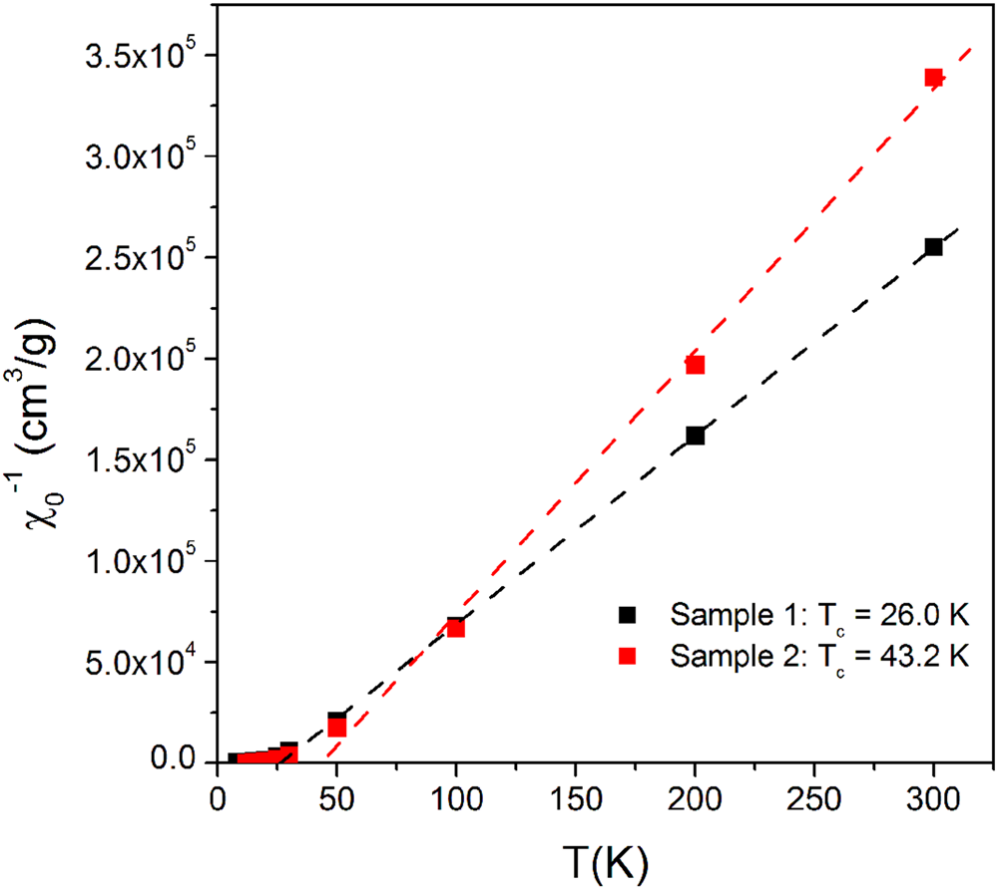
Reciprocal of the initial magnetic susceptibility as a function of temperature in samples 1 and 2.

Usually, in standard nanoparticulate systems the FC/ZFC magnetization curves keep distinctly nonzero values even up to high temperatures; in our materials instead, these curves quickly reduce to vanishingly small values with increasing T, the magnetic signal becoming virtually undetectable above about 35 K (Fig. 4(a)). This behavior can be understood by looking at the reciprocal of the initial magnetic susceptibility χ_0_^−1^ (plotted in Fig. 8) as a function of temperature for both samples. A linear behavior of χ_0_^−1^ with a positive intercept on the x-axis is observed at high temperatures. This behavior corresponds to the linear, reversible M(H) curves measured at high temperatures (Figure S1) and is associated with a purely paramagnetic behavior of the individual Ni^2+^ ions well described by a Curie-Weiss law. Using the number of magnetic ions (per unit mass) given by EDX measurements (see previous subsection), the slope of χ_0_^−1^ corresponds to an effective magnetic moment per Ni ion μ_eff_ = 2.89 × 10^−20^ emu = 3.12 μ_B_ in sample 1 and μ_eff_ = 2.27 × 10^−20^ emu = 2.45 μ_B_ in sample 2. These figures are similar to the μ_eff_ values currently found on Ni^2+^ ions in a variety of nanostructures^47–49^ and may indicate incomplete quenching of the orbital magnetic moment. As a consequence, the number of aligned Ni^2+^ spins taking part in coherent QTM is n_AS_ ≅ 32 in sample 1 and n_AS_ ≅ 15 in sample 2.

The Curie temperatures obtained by the fits are T_C1_ = 26 K and T_C2_ = 43 K for the two materials. The observation of a Curie temperature (instead of a Néel temperature) indicates that the interactions among individual spins in the ultra-small particles are predominantly ferromagnetic. However, this magnetically ordered state is not particularly robust, breaking down into statistically independent ionic spins above T_C_. The above reported difference in T_c_ is in good agreement with the observation of a higher amount of lamellar sub-nanoparticles containing Ni^2+^ ions in sample 2.

As a matter of fact, the lamellar sub-nanoparticles observed in sample 2 are likely to contain a larger total number of Ni^2+^ ions than the magnetic sub-nanostructures of sample 1(see Fig. 1); this seems in contradiction with the results of the fitting procedure used to analyze the S_V_(H) curves of Fig. 5 (bottom panel) giving n_AS_ ≅ 32 and n_AS_ ≅ 15 for the total number of magnetic units involved in QTM for samples 1 and 2, respectively. In particular, the value n_AS_ ≅ 15 is significantly smaller than expected in lamellar sub-nanoparticles of the size observed in sample 2 (see Fig. 1). This difficulty can be removed remarking that in a lamellar structure the interplanar magnetic interactions of dipolar nature^50^ are much weaker than the intraplanar exchange interactions, as proven by the results of magnetic measurements on nanometer-sized nickel phyllosilicates^50^. As a consequence, it is conjectured that the weak interplanar magnetic interactions are not able to guarantee the coherent tunneling of all spins present in lamellar sub-nanoparticles, and that the magnetic units responsible for QTM in sample 2 are not the whole sub-nanoparticles but the single lamellae present in them.

The conclusion that not all spins in these nanostructures perform the coherent tunneling through the barrier is strengthened by a comparison between the magnetic moments involved in tunneling as obtained from relaxation measurements and the values estimated from a Langevin-function fit of the isothermal magnetization curves taken in the superparamagnetic region, i.e., just above the blocking temperature (Figure S3 in the Supplementary Information). The results of such a fit are: μ_Lang_ ≅ 180 μ_B_ in sample 1 (at T = 8 K) and μ_Lang_ ≅ 390 μ_B_ in sample 2 (at T = 12 K). These values are definitely larger than the ones obtained from relaxation measurements (μ = 100 μ_B_ and μ = 36 μ_B_ respectively). It can be concluded that in sample 1 a large part of the overall nanostructure magnetization is involved in tunneling; in sample 2, the ratio μ/μ_Lang_ is much smaller, in agreement with the previous hypothesis that the magnetic units responsible for QTM are the single lamellae instead of the whole nanostructure.

The following overall picture of the magnetic states of the investigated systems emerges: at high temperature, i.e., above T_C_ the units responding to the magnetic field are the individual Ni^2+^ ions present inside the ultra-small particles decorating the larger SiO_2_ nanoparticles (Figure S4(a)); the experimental evidence indicates that the interaction among these ions is ferromagnetic and leads to the onset of a magnetic order (extending over the particle) below T_C_; a “superspin” S is now associated with each sub-nanostructure or each sub-nanoparticle lamella which therefore exhibits a net magnetic moment. Between T_B_ and T_C_, the ultra-small magnetic units are in the superparamagnetic regime (Figure S4(b)). According to the classical picture, at temperatures lower than T_B_ the magnetic units would become blocked (Figure S4(c)); however, features and properties of the significant relaxation observed below T_B_ are fully compatible with the onset of phonon-assisted, resonant tunneling of magnetization (Figure S4(d)).

## Conclusions

Magnetic sub-nanostructures and/or sub-nanoparticles decorating SiO_2_ nanospheres were obtained by adding Ni^2+^ ions to the preformed nanospheres in two different ways, resulting in different ultra-small, magnetically active units: in one case, sub-nanostructures of few nanometers, almost amorphous; in the other case, a denser texture of elongated, lamellar sub-nanoparticles (very thin along the [001] direction) at the nanosphere surface.

In spite of their different morphologies on the nanometer scale, the two heterogeneous nanomaterials under study basically exhibit closely similar magnetic states at comparable temperatures, the differences being naturally ascribed to the different aspect ratio of the magnetic sub-nanostructures/sub-nanoparticles decorating the SiO_2_ nanospheres. In both materials, a peculiar behavior emerges at the lowest investigated temperature, indicating the onset of quantum effects. The distribution of magnetically active sub-nanostructures on the surface of larger diamagnetic spheres turns out to be particularly effective in reducing magnetic interactions among them, making both systems ideal playgrounds to study their magnetic states, including quantum effects having a prospective interest for applications in the information technology area.

## Materials and Methods

All reagents and solvents used for materials preparation (namely, tetraethylorthosilicate, aminopropyltriethoxysilane, Triton X-100, ammonia, cyclohexane, n-hexanol, ethanol, acetone, NiCl_2_) are high purity Sigma-Aldrich products and were used as received.

### Preparation of nanoparticles

Bare SiO_2_ nanosphere, considered for the sake of comparison in morphological and structural investigations, were prepared as follows: a microemulsion was prepared by mixing 75 ml of cyclohexane, 18.85 g of Triton X-100, 18 ml of *n*-hexanol and 5.4 ml of MilliQ H_2_O and then equilibrated by magnetic stirring for 30 minutes at room temperature. The formation of silica particles was started by addition of 1.0 ml of tetraethylorthosilicate (TEOS) and 0.7 ml of NH_3_ (28–30%) and continued at R.T. under magnetic stirring. for 16 hours, time necessary for the accomplishment of reactions resulting in the formation of SiO_2_ NPs^51^. Nanoparticles were finally extracted from the reaction mixtures by centrifugation (10000 rpm, 20 minutes) and washed twice in ethanol and several times in MilliQ H_2_O by re-suspension/centrifugation cycles.

The addition of Ni^2+^ to preformed SiO_2_ NPs was carried out in two different ways. Because of the beneficial effect of amino groups in the dispersion of these cations on silica surface^10^, in the first case, 0.3 ml of a solution containing aminopropyltriethoxysilane (APTS, 3.73e-3 moles) and NiCl_2_ (3.4e-4 moles) were added to the microemulsion already reacted for 16 h in order to form SiO_2_ nanoparticles. The so obtained material will be hereafter referred to as sample 1. For the preparation of the second type of nickel containing material (hereafter, sample 2), only the water solution of NiCl_2_ was added to the microemulsion with preformed SiO_2_ NPs. In both cases, the systems were kept under stirring at R.T. for further 24 hours after Ni addition and then nanoparticles were recovered as reported for bare SiO_2_ NPs.

### Characterization techniques

*High resolution transmission electron microscopy* (HRTEM), coupled with *energy dispersive X-ray spectroscopy* (EDX). Micrographs of nanoparticles were acquired using a 3010 Jeol instrument operated at 300 kV. A droplet of each sample suspended in MilliQ H_2_O (1.0 mg mL^−1^) was spread on a copper grid coated with a lacey carbon film and then water was slowly evaporated to limit particle agglomeration. The mean particle diameter was calculated as d_m_ = Σd_i_n_i_/Σn_i_ (n_i_ = number of particles of diameter d_i_) by measuring the size of ca. 300 particles. The obtained results were expressed as d_m_ ± stdv. The instrument was equipped with an Oxford INCA Energy TEM 200 EDX analyzer. Obtained data were quantitatively treated using the Oxford INCA Microanalysis Suite software. For chemical analysis, EDX spectra produced by NPs present in 10 fields of view (magnification: 30 kx), each of them containing the image of 20–30 NPs, were collected, and the resulting data averaged.

*X-ray powder diffraction* (XRPD) patterns of each sample were collected on a PANalyticalX’Pert PRO instrument operating with Cu Ka radiation (1.54 Å), generated at 45 kV and 40 mA.

*Diffuse reflectance* (DR) *UV-Vis-NIR* spectra were collected with a Varian Cary 5000 spectrophotometer, equipped with an integrating sphere with inner coating in Spectralon®, also used as reference. Aliquot of the samples were inserted in a conventional cell for loose powders provided by Varian, basically constituted by a spring acting on a metal disk pressing the sample on an optical quartz window. Samples were dosed in order to attain powder layers ticker than 2 mm, in order to avoid possible contribution to reflection of the metal disk on their back. This minimum thickness was established by assessing the absence of any transmission when filling with samples a cuvette with an optical path of 2 mm.

Magnetic measurements were performed in the 2–300 K range using a a Lot Quantum Design MPMS3 SQUID magnetometer with maximum field H_M_ = 70 kOe. The rate of change of H in isothermal magnetization loops was of 2.5 Oe/s in the central region (|H| < 1 kOe) and higher outside. FC/ZFC magnetization curves were measured under a field of 50 Oe with a rate of 2 K/min. AC susceptibility measurements were conducted in the same SQUID magnetometer in the frequency range 0.1–1 × 10^3^ Hz under an applied field of about 1 Oe in the temperature interval 2- 50 K. All relaxation experiments were performed after an initial time lapse related to the dynamic response of the measuring setup, whose reaction/stabilization times are: a) the cooling rate between 10 and 2 K is 10 K/min; any change of the set temperature and the ensuing thermal stabilization are completed in less than 5 minutes; the temperature stability in isotherms is ±0.5%; b) the maximum field change rate is 700 Oe/s; the field resolution is 0.33 Oe. The magnetic relaxations at T = 2 K were measured in the SQUID magnetometer by keeping the magnetic field fixed at the selected value for a time up to 3600 s. The DC initial magnetic susceptibility was obtained from the magnetization loops. All results were corrected for the diamagnetic contribution of the SiO_2_ nanoparticles.

### Data availability statement

All data are available at paolo.allia@polito.it (magnetic data) giamnario.martra@unito.it (structural data).

## Electronic supplementary material

Supplementary Information
