# Supplementary material for: Magnetic states of nanostructures containing Ni2+ ions at the surface of SiO2 nanospheres
Source: Sci Rep. 2017 Sep 7;7:10822. doi: 10.1038/s41598-017-11394-8 (PMC5589877; doi:10.1038/s41598-017-11394-8)
Supplement: Supplementary file 1 — Supplementary Information [file 41598_2017_11394_MOESM1_ESM.pdf]

SUPPLEMENTARY INFORMATION

Magnetic states of nanostructures containing Ni^{2+} ions at the surface of SiO_2 nanospheres

Author list:

Gabriele Barrera, Gabriele Alberto, Paola Tiberto, Gianmario Martra, Paolo Allia[*]

*Address correspondence to *paolo.allia@polito.it*

1.Additional Results

The magnetization of the two samples is reported in Fig. S1 as a function of magnetic field at different temperatures between 2 and 300 K. The quantity is defined as the measured magnetic moment per unit mass of the sample, whose weight is dominated by the diamagnetic silica NPs. In both cases, a linear, paramagnetic behavior is observed at high temperatures (above 50 K); magnetic hysteresis appears below 8 K (sample 1) and 12 K (sample 2).

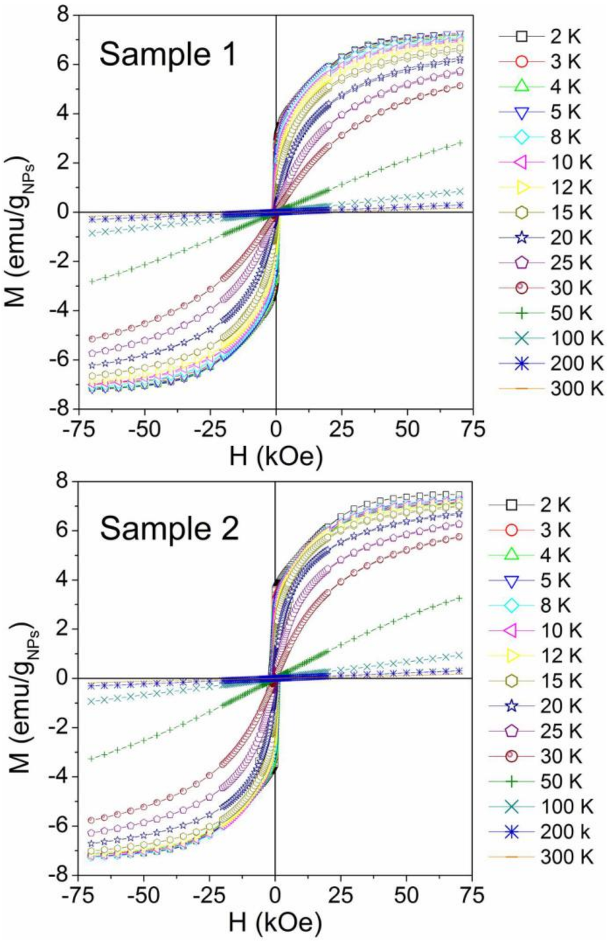

Figure S1. Isothermal magnetic hysteresis loops for samples 1 and 2.

The imaginary part of the AC susceptibility $\chi''(T)$ is shown in Figure S2 (top panel) for sample 2 (a closely similar behavior is found in sample 1). The temperature of the maximum of $\chi''(T_{max})$ increases with increasing frequency. When the logarithm of measurement frequency is plotted as a function of $1/T_{max}$ the data are well aligned on a straight line (Figure S2, bottom panel).

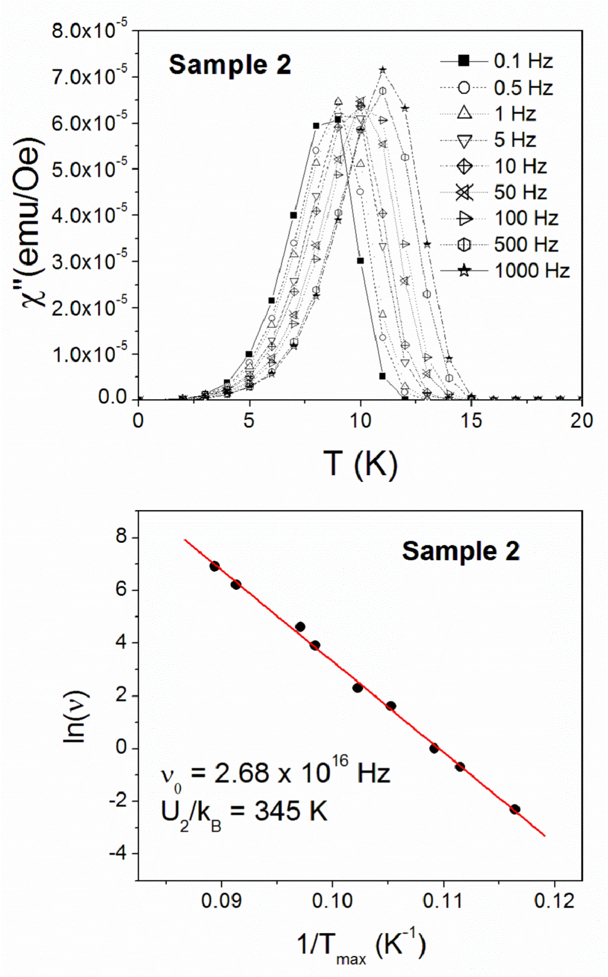

Figure S2. Top panel: Imaginary part of the AC susceptibility (χ'') as a function of temperature at different frequencies in sample 2; bottom panel: symbols: logarithm of measurement frequency as a function of the reciprocal of the temperature of the maximum of χ''; line: best fit to the Arrhenius law.

2. Magnetic viscosity of an assembly of independent quantum nanoparticles with random anisotropy axes

A model is introduced to describe the magnetic viscosity displayed by a three-dimensional system of quantum nanoparticles evolving towards equilibrium through crossing of an anisotropy energy barrier via coherent quantum tunneling (both resonant and phonon-assisted). The anisotropy axes are assumed to point along random directions in space.

When the magnetic field H_A points along a direction defined by the angle ϕ with respect to the easy axis of a NP (the z-axis in a local reference frame) the magnetic field H_A can be considered as split in one contribution parallel to the easy axis ($H_{A//} = H_A \cos\phi$) and one perpendicular to it ($H_{A\perp} = H_A \sin\phi$); in the first case, the energy is given by $E = Usin^2\theta - \mu H_{A//} cos\theta$, where U is the anisotropy barrier, θ is the angle between M and the z-axis; in the second case, $E = Usin^2\theta - \mu H_{A\perp} sin\theta$; this implies that $H_{A\perp}$ does not make the double well asymmetric; it just decreases the height of the barrier and displaces both minima towards the perpendicular direction ($\theta = \pi/2$); as a result, no imbalance of N_1 and N_2 is produced by $H_{A\perp}$. The behavior of S_V with field H at fixed temperature for a system of independent particles with randomly distributed easy-axis directions is obtained considering for each angle ϕ the evolution of the fractions $N_{1\phi}$, $N_{2\phi}$ of particles in the double wells starting from the off-equilibrium state induced by the applied field H_A .

We consider nanoparticles of total spin S and magnetic moment $\mu = 2\mu_B S$ described as double-well systems with anisotropy barrier U.

a) Magnetic field aligned with the easy axis direction (ϕ=0)

The double well is symmetric when H = 0. When a magnetic field H_A is applied at the temperature T_A the well becomes asymmetric with $E_1 < E_2$ (see Figure 6(a) of main text) The energy difference is $E_2 - E_1 = 2\mu H_A$. Defining $\beta = \frac{2\mu H_A}{k_B T_A} \gg 1$, the equilibrium population in each well is

$$N_{01} = N_0 \frac{1}{1+e^{-\beta}} \approx N_0$$

$$N_{02} = N_0 \frac{e^{-\beta}}{1+e^{-\beta}} \ll N_0$$

N_0 being the total number of double-well systems with easy axis aligned with the field direction.

At the temperature $T \ll T_A$, immediately after setting the field to $H < H_A$, the magnetization takes the off-equilibrium value:

$$M_{0i} = (N_{01} - N_{02})_i\, \mu = \frac{1-e^{-\beta}}{1+e^{-\beta}}\, \mu$$

the final (equilibrium) value being instead:

$$M_{0f} = (N_{01} - N_{02})_f\, \mu = \frac{1-e^{-\alpha H}}{1+e^{-\alpha H}}\, \mu$$

where $\alpha = \frac{2\mu}{k_B T}$. Note that when H = 0 the final value of M_0 is expected to be zero, corresponding to $N_{01} = N_{02} = N_0/2$. If the logarithmic relaxation of M(t) is related to the presence of a flat distribution of energy barriers p(U) in the interval $U_1 \leq U \leq U_2$ with the

associated distribution of relaxation times $p(\tau)$ in the interval $\tau_1 \leq \tau \leq \tau_2$ at the temperature T, the magnetic viscosity $S_V(T,H)$ is:

$$S_V(T,H)=\frac{1}{\ln\frac{\tau_2}{\tau_1}}\left(1-\frac{M_{0f}}{M_{0i}}\right)=\frac{1}{\ln\frac{\tau_2}{\tau_1}}\left(1-\frac{\frac{1-e^{-\alpha H}}{1+e^{-\alpha H}}}{\frac{1-e^{-\beta}}{1-e^{-\beta}}}\right)$$

When $\beta>>1$, $S_V(T,H)\cong\frac{1}{\ln\frac{\tau_2}{\tau_1}}\left(1-\frac{1-e^{-\alpha H}}{1+e^{-\alpha H}}\right)=\frac{2}{\ln\frac{\tau_2}{\tau_1}}e^{-\alpha H}$.

b) Magnetic field taking an arbitrary direction ϕ_n with respect to the easy axis ($-\pi/2 \leq \phi_n \leq \pi/2$)

The field's components parallel and perpendicular to the nanoparticle's easy axis are: $H_{//} = H \cos \phi_n$, $H_{\perp} = H \sin \phi_n$. The distribution of population in the two wells is determined by $H_{//}$, the component $H_{\perp}$ not contributing to the asymmetry of the double well; therefore:

$$N_{\varphi_n 1}=N_{\varphi_n}\frac{1}{1+e^{-\beta\cos\varphi_n}}$$

$$N_{\varphi_n 2}=N_{\varphi_n}\frac{e^{-\beta\cos\varphi_n}}{1+e^{-\beta\cos\varphi_n}}$$

$N_{\phi n}$ being the total number of double-well systems with easy axis taking an angle ϕ_n with respect to the field direction. Usually $\beta \cos \phi_n >> 1$ and $N_{\phi n1} >> N_{\phi n2}$; however $N_{\phi n1} = N_{\phi n2} = N_{\phi n}/2$ when $\phi_n \rightarrow \pi/2$.

When the temperature is lowered to $T << T_A$ and H is decreased to $H < H_A$, the starting magnetization in the direction of the field is:

$$M_{\varphi_n i} = \left(N_{\varphi_n 1} - N_{\varphi_n 2}\right)_i \mu \cos\varphi_n + \varepsilon_n \sin\varphi_n$$

where $\varepsilon_n = \frac{N_{\varphi n}\mu^2 H}{2U} \ll 1$ is the contribution to the magnetization *in the field direction*, U being the anisotropy barrier of the double well. The final (equilibrium) magnetization is:

$$M_{\varphi_n f} = \left(N_{\varphi_n 1} - N_{\varphi_n 2}\right)_f \mu \cos\varphi_n + \varepsilon_n \sin\varphi_n$$

c) System of nanoparticles with randomly oriented anisotropy axes

Let us consider a system of nanoparticles with randomly oriented anisotropy axes; it is assumed that the number of nanoparticles is evenly distributed ($N_{\phi n}$ = const.). Dividing the interval $(-\pi/2, +\pi/2)$ into $\mathcal{N}$-1 intervals ($\mathcal{N}>>1$), the initial value of the overall magnetization along H is:

$$M_i = \frac{1}{\mathcal{N}}\left[\mu \sum_{n=1}^{\mathcal{N}} \frac{1-e^{-\beta\cos\varphi_n}}{1+e^{-\beta\cos\varphi_n}} \cos\varphi_n + \varepsilon \sum_{n=1}^{\mathcal{N}} \sin\varphi_n\right] = \frac{1}{\mathcal{N}}\left[\mu \sum_{n=1}^{\mathcal{N}} \frac{1-e^{-\beta\cos\varphi_n}}{1+e^{-\beta\cos\varphi_n}} \cos\varphi_n\right]$$

In fact, the second sum gives zero by symmetry reasons, ε being a constant quantity if $N_{\phi n}$ = const. By analogy, the final value of the overall magnetization is:

$$M_f = \frac{1}{\mathcal{N}} \left[\mu \sum_{n=1}^{\mathcal{N}} \frac{1\text{-}e^{-\alpha H \cos\varphi_n}}{1+e^{-\alpha H \cos\varphi_n}} \cos\varphi_n \right]$$

and the magnetic viscosity takes the form:

$$S_V(T,H) = \frac{1}{\ln\frac{\tau_2}{\tau_1}} \left(1\text{-}\frac{M_f}{M_i}\right) = \frac{1}{\ln\frac{\tau_2}{\tau_1}} \left(1\text{-}\frac{\sum_{n=1}^{\mathcal{N}} \frac{1\text{-}e^{-\alpha H \cos\varphi_n}}{1+e^{-\alpha H \cos\varphi_n}} \cos\varphi_n}{\sum_{n=1}^{\mathcal{N}} \frac{1\text{-}e^{-\beta \cos\varphi_n}}{1+e^{-\beta \cos\varphi_n}} \cos\varphi_n}\right) \qquad (1)$$

In particular, when H = 0 one has $S_V(T,0) = \frac{1}{ln\frac{\tau_2}{\tau_1}}$ because M_f=0.

3. A pictorial representation of the different magnetic states in magnetic sub-nanoparticles

The magnetic states of the investigated systems are summarized in Fig. S3. Classical blocking (case c) is actually overcome by QTM (case d).

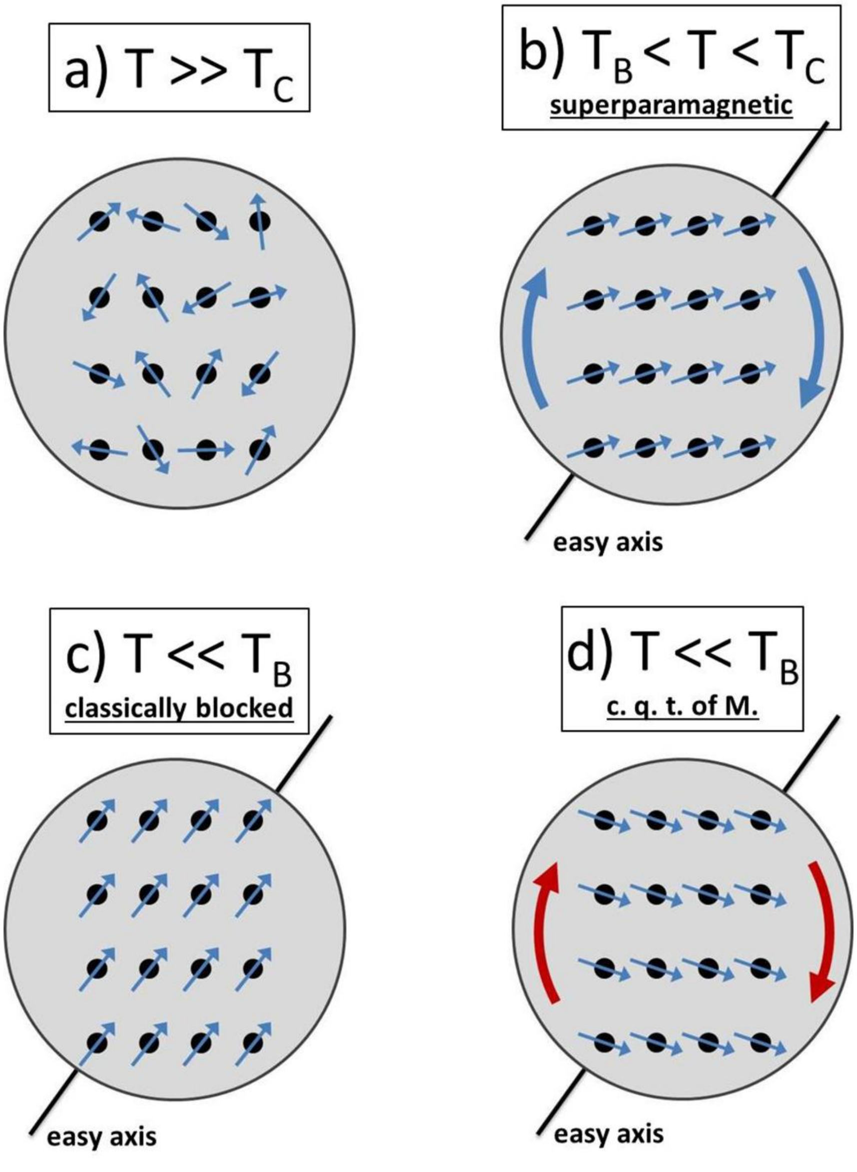

Figure S4. Overall picture of arrangement and behavior of Ni^{2+} spins in samples 1 and 2 at different temperatures.
